# Characterization of heavy metal contamination in groundwater of typical mining area in Hunan Province

**DOI:** 10.1038/s41598-024-63460-7

**Published:** 2024-06-06

**Authors:** Wenjie Hao, Huan Liu, Shuli Hao, Kuanzhen Mao

**Affiliations:** 1 Center for Hydrogeology and Environmental Geology, China Geological Survey, Tianjin, 300304 China; 2 Technology Innovation Center for Geological Environment Monitoring, MNR, Baoding, 071051 China; 3 School of Precision Instruments and Optoelectronics Engineering, Tianjin University, Tianjin, 300072 China; 4 Qingdao Geological and Mineral Geotechnical Engineering Co., Ltd, Qingdao, 266101 China

**Keywords:** Groundwater, Heavy metals, Statistical characterization, Pollution contribution, Correlation, Environmental sciences, Hydrology

## Abstract

Heavy metal pollution in mining areas is a major cause of groundwater contamination, characterized by high toxicity, difficult degradability, and easy accumulation, and the source of pollution is not easily identified. Relying on the results of groundwater quality analysis tests in a typical mining area, this paper uses the SPSS 18.0 statistical analysis model to analyze the statistical characteristics of different indicator factors in the antimony mining area. The conclusions play a crucial role in implementing health and safety measures for the mining area and its surrounding residents. The statistical study results show that Mn, Se, As, and Sb are closely related to human mining activities and are polluted to varying degrees; the principal component analysis model indicates that the upstream monitoring points 26#, 22#, and 25# in the mining area groundwater are less polluted. The five monitoring points with a comprehensive principal component F > 1 are all located within the range of the metal mine cluster, indicating that the groundwater in the mining area is particularly sensitive to the impact of anthropogenic mineral extraction. This research summarizes the hydrogeological and geochemical statistical characteristics of the groundwater in the mining area, providing a reference for groundwater pollution risk diagnosis, ecological restoration, and heavy metal pollution prevention and control in this and similar mining areas.

## Introduction

The Problem of Groundwater Pollution has become a major concern for governments, businesses, and the public^1^. There is a growing awareness among people, and water quality safety has increasingly become an important issue for national sustainable development and ensuring public health^2–5^. Therefore, conducting relevant research holds significant importance for the ecological environment construction and protection of regional groundwater resources^6^. Metal mining areas are one of the main sources of groundwater pollution, the cumulative effect of these heavy metal elements in the human body poses significant potential risks to the health of regional populations, causing severe harm to human organs^7–9^. The main source of these heavy metals is the mining activities that generate enormous value. The resulting tailings, dust, waste rocks, and wastewater from mining operations can lead to the diffusion and infiltration of heavy metals into soil and groundwater, causing severe pollution to groundwater bodies, thus resulting in irreparable harm to human health^10^.

The application of mathematical and statistical analysis models in the analysis of groundwater pollution characteristics and source resolution is becoming increasingly widespread. In the study area, the antimony mining district features a variety of minerals, leading to a diverse range of heavy metal pollutants. Thus, mathematical and statistical analysis of groundwater in the mining area is particularly important. In the mining process, the groundwater quality is consistently susceptible to pollution. Conducting statistical analysis on the groundwater of typical mining areas helps ensure the health and safety of the residents, and aids in understanding the distribution characteristics of heavy metal pollution in the groundwater, the main sources of pollution, and the contribution rates of major pollutants. This research enables relevant personnel to better understand the state of groundwater pollution in the mining area, aimed at providing a scientific basis for mineral extraction and utilization, planning, and the protection and management of groundwater. It also offers references for the formulation of public health risk control in the mining area and groundwater pollution treatment and restoration plans, thereby facilitating the development of effective measures to reduce health risks to residents.

## Overview of the study area

### Socioeconomic overview of the study area

The investigation area is situated in the northwest of Lengshuijiang City, Hunan Province, encompassing the Mining Township, the Xikuangshan Administration, and the border area of Zhonglian Township. It lies approximately 13 km south of Lengshuijiang City, with geographical coordinates ranging from 111°25′47″ to 111°31′22″ east longitude and 27°49′28″ to 27°43′05″ north latitude. The antimony mining industry in this region began production in 1897 and has a history of over a century. Mineral production and processing serve as the primary economic sources for the local government and residents, earning the area the titles of “World Capital of Antimony” and “Coal Sea of Jiangnan”^11,12^.

Within this mining area, two large ore deposits, one medium-sized ore deposit, and three small-sized ore deposits have been identified. The antimony mineral field in the northern Xikuangshan area is the concentration zone for antimony mineral production, with an accumulated confirmed reserve of 26.265 million tons (metal reserve of 855,202 tons). The reserve is exceptionally abundant, ranking first in the nation and representing the world's largest antimony mineral field^13,14^. The total population residing in the mining area is approximately 17,100, comprising approximately 15,000 urban residents and around 2100 rural villagers^15,16^.

Over the past hundred years of mining operations in the Xikuangshan antimony mining area, the substantial generation of “three wastes”—waste gas, waste residue, and waste water—has caused severe environmental damage to local soil and groundwater. Consequently, it has posed varying degrees of health risks to the local residents^17,18^. As the subject of this investigation, the Xikuangshan mining area demonstrates typicality and representativeness, thus offering outcomes of considerable representational significance in the research.

### Hydrogeological conditions

#### Fault zone permeability and aquifer characteristics

The fault zones within the area, including the F75, F72, F3 of the North-Northeast group, and the F19, F17, F104 of the North-Northwest group, constitute significant and mechanically similar normal faults within the ore deposit. Due to the surrounding rocks being predominantly composed of argillaceous limestone, sandstone, and shale, the development of karstification is not favored. The fractured zones within the fault zones consist mainly of fractured mudstones, sandstones, and limestone blocks with a dense cementation, which impedes groundwater movement and storage. Drilling observations within these fault zones did not reveal any water seepage. Water injection tests conducted via boreholes demonstrated minimal water injection rates (0.0014L/S·m), particularly within the main F75 fault, where 14 cross veins in the midsections of tunnels 7, 9, 11, and 13 displayed dry fault surfaces without any indications of groundwater activity. These fault zones are entirely filled with fragmented rocks such as sandstone, limestone, and shale. However, smaller secondary fault zones and fissures were observed in the tunnel exposures, facilitating water storage within the ore deposit, primarily channeling fractured water from silicified limestone and silicified rock zones. These secondary faults trend in the northeast and northwest directions, with an inclination exceeding 60°, extending several meters to tens of meters in length, and possessing a maximum width of approximately 1.5 m. The predominant lithology comprises silicified limestone, followed by limestone, with flow rates ranging from 0.01 to 0.5L/S. Based on the above description, the fault zones within this mining area exhibit relatively poor water-bearing and water-conducting properties.

#### Groundwater flow status

The Xikuangshan mining area is situated at the hydrological watershed of the aquifer system. To the east, the Yunxi biotite granite forms a hydrological barrier, while the F75 fault acts as the western hydrological boundary. Consequently, it creates a north–south flowing aquifer unit, effectively isolating the ore deposit from the regional groundwater. Moreover, a local hydrological watershed exists in the central part of the study area, positioned at Qilijiang, resulting in independent flow divisions between the northern and southern mines. Additionally, surface water bodies distributed within the ore deposit are all relatively small.

As a result, the groundwater in the ore deposit primarily relies on infiltration from atmospheric precipitation for recharge. Natural features such as exposed rock cavities, fractures, and old workings become recharge zones for the aquifer. A few unclosed boreholes and surface silicified limestone fractured zones also contribute to the aquifer recharge. Consequently, the overall recharge area of the aquifer is relatively limited.

The groundwater flow in the aquifer predominantly exists in the form of limestone fissures, karst caves, interlayer fractured zones, and fractured fissures in silicified limestone. Generally, the groundwater flows from the Qilijiang watershed in the central part of the study area, discharging southwards and northwards from the study area. The runoff distance is relatively short. The primary discharge method is in the form of springs, with only a minimal portion discharged through faults. Extensive mining activities in the Xikuangshan area have led to the formation of artificial discharge zones in deeper mining regions.

The dynamics of the ore deposit groundwater are primarily characterized by significant fluctuations in water level and quantity. Since it is predominantly replenished by rainfall, variations in groundwater dynamics are closely related to precipitation. Seasonal changes are evident, with groundwater levels rapidly rising during the rainy season, leading to a sharp increase in flow rates. For instance, observation at Spring No. 7 recorded a flow rate of 2.129L/S before rainfall, surging to 14.13L/S within the initial 30 min of rainfall. The monthly amplitude of groundwater levels is generally around 2.40 m, with an average annual amplitude of 7.6 m.

Mining activities in the area have moderately impacted the underground aquifer due to groundwater extraction. However, water scarcity has not been observed, and there have been minimal occurrences of wells or springs running dry. Overall, the impact on regional groundwater balance remains relatively slight.

## Sample collection and testing methods

### Principles of sampling point layout

Based on the “Technical Specifications for Groundwater Environmental Monitoring” (HJ164-2020), “Technical Guidelines for Site Environmental Monitoring” (HJ25.2–2014), “Sanitary Standards for Drinking Water” (GB5749-2006), “Groundwater Quality Standards” (DZ/T0290-2017), as well as the “Technical Regulations for National Integrated Water Resources Planning,” the following principles for sample point layout were formulated:

1. The southern and northern mining areas belong to different hydrogeological units. Accordingly, sampling points were arranged based on the distinct hydrogeological conditions of these independent units.
2. Considering the distribution of pollution sources such as leachate from mine waste, ore washing wastewater, and pit drainage, water quality monitoring points were set up at the groundwater recharge, flow, and discharge sections in each hydrogeological unit of the southern and northern areas. The spacing between points generally ranged from 150 to 200 m.
3. In addition to the existing monitoring wells, domestic wells, and springs within the mining area, additional groundwater quality monitoring points were selectively added and adjusted based on the actual exposure of groundwater and the conditions of recharge, flow, and discharge. The placement of monitoring points was adjusted according to the specific conditions, avoiding uniform distribution.

### Layout of monitoring network

1. The controlled area of the groundwater quality pollution monitoring network within the mining area covers an area of 30km^2^.
2. In the mining area, a total of 26 groundwater monitoring points and 4 surface water quality monitoring points have been established.

**Figure 1:**
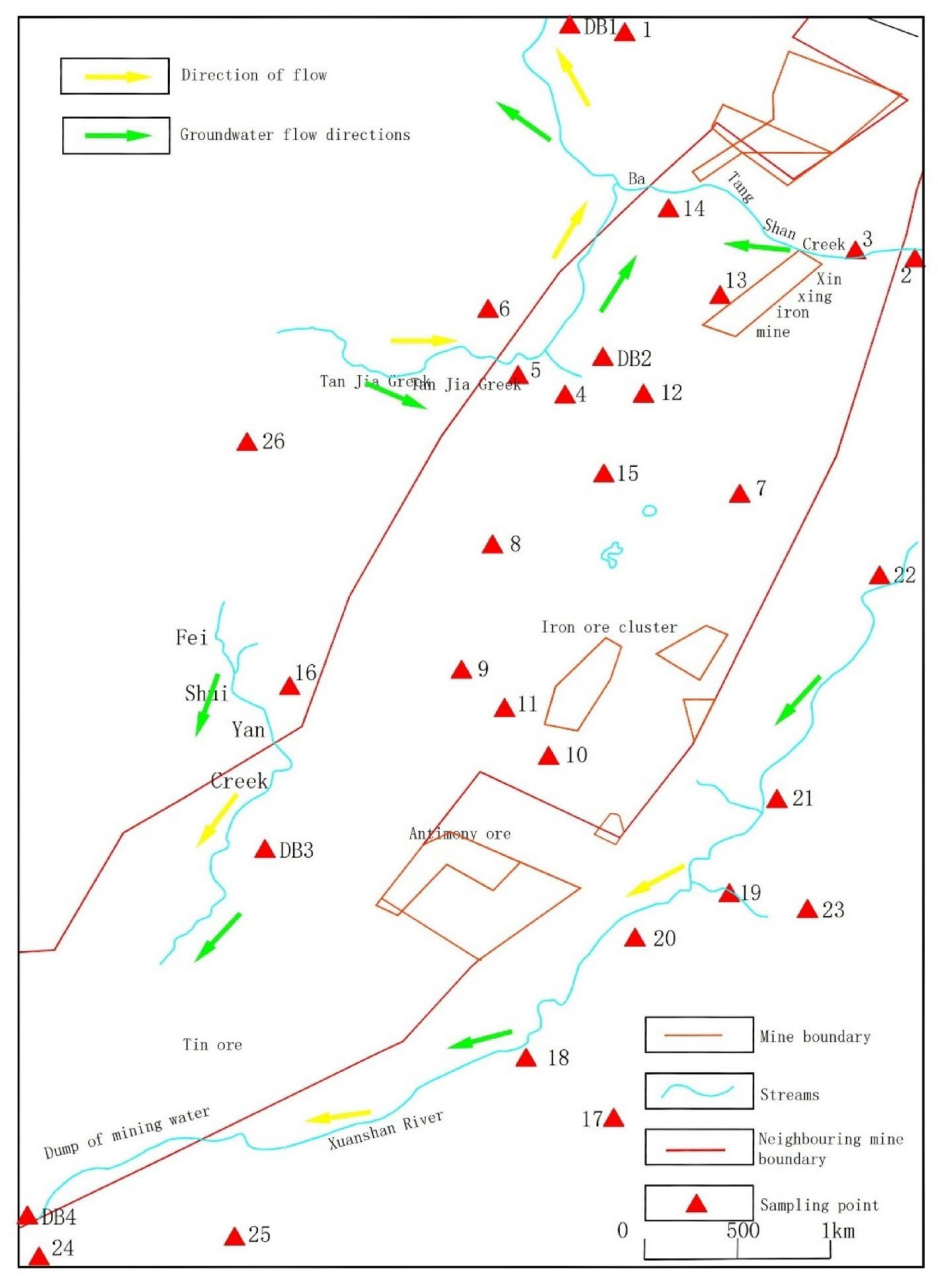
The sampling point distribution in the study area.

This research presents a distribution map of the water quality monitoring points in the study area, as shown in Fig. 1.

### Principles for selecting heavy metal evaluation indicators

Multiple characterization factors need to be considered to achieve the groundwater functional assessment’s objective. However, the selection of characterization indicators varies widely, and it's not advisable to mechanically or indiscriminately include all factors in the evaluation index system. Doing so might dilute the role of dominant factors, affecting the accuracy of the assessment results. Therefore, it’s essential to make an optimal selection of evaluation factors based on principles such as: the principle of dominance, measurability, operability, comprehensiveness, flexibility, among others.

The mineral composition in the study area is relatively straightforward, with relatively simple chemical components. Apart from the primary element antimony (Sb), associated elements include As, Hg, Ag, Cu, Pb, Sn, V, Zn, Mo, Ga, and B. Following the classification system established by the International Agency for Research on Cancer (IARC) and the World Health Organization (WHO) for assessing the reliability of the carcinogenicity of chemical substances, essential trace elements for the human body in the associated elements within the study area include Mo, Co, Mn; harmful substances comprise As, Ba, Sb; carcinogenic substances include As, non-carcinogenic substances consist of Mn, Se, and potentially carcinogenic substances encompass Co, Sb.

Considering the aforementioned principles and the evaluation objectives of this study, the heavy metals Mo, As, Co, Mn, Ba, Sb, and Se were chosen as evaluation indicators.

### Sampling and testing methods

#### Groundwater sampling and preservation

In accordance with the “Standard Examination Methods for Drinking Water” (GB5749-2006, GB/T5750.1-5750.13-2006), containers were acid-washed or alkali-washed as specified before sampling, followed by rinsing with distilled water. The sampling instruments were rinsed with the source water at least three times before sampling. Non-disposable groundwater sampling equipment was used, requiring pre- and post-sampling cleaning. Wastewater generated during the cleaning process should be collected and disposed of properly. For sample bottles without preservatives, they were rinsed with the water to be sampled two to three times before groundwater sampling. Samples containing heavy metals such as As, Mn, Sb were collected in 250 ml polyethylene plastic bottles, adjusted to pH less than 2 with nitric acid, and analyzed within 10 days. The frequency of this sampling was once and the sampling period was two days.

#### Groundwater sample testing methods

Adhering to the requirements of the “Standard Examination Methods for Drinking Water” (GB/T5750.6-2006) and “Water Quality-Determination of Barium” (HJ 602-2011) for various indicators, different instruments were equipped to ensure the quality of sample analysis based on the quality level of different analysis methods. Main testing instruments included gas chromatography-mass spectrometer, atomic absorption spectrophotometer, and atomic fluorescence spectrometer, among others. The standard testing methods used for various detection components are detailed in Table 1.

**Table 1:** Test method of heavy metal evaluation index factors for groundwater samples.

Elemental	Standard	sample size	Methodologies
As	GB/T 5750.6–2006	1L	Standard Test Methods for Drinking Water METAL INDICATORS (6.1) Hydride atomic fluorescence method
Se	GB/T 5750.6–2006		Standard Test Methods for Drinking Water METAL INDICATORS (7.1) Hydride atomic fluorescence method
Sb	GB/T 5750.6–2006		Standard Test Methods for Drinking Water METAL INDICATORS (19.1) Hydride atomic fluorescence method
Co	GB/T 5750.6–2006		Standard Test Methods for Drinking Water METAL INDICATORS (14.1) Flameless atomic absorption spectrophotometry
Mo	GB/T 5750.6–2006		Standard Test Methods for Drinking Water METAL INDICATORS (13.1) Flameless atomic absorption spectrophotometry
Mn	GB/T 5750.6–2006		Standard Test Methods for Drinking Water METAL INDICATORS (4.2) Flame atomic absorption spectrophotometry
Ba	HJ 602–2011		Water quality Determination of barium Graphite Furnace Atomic Absorption Spectrophotometry

### Sample collection, testing quality control, and assurance measures

#### Quality control in the sampling process

Accordance with “Technical Guidelines for Water Quality Sampling” (HJ 494-2009), “Technical Specifications for Groundwater Environmental Monitoring” (HJ 164-2020), and “Technical Regulations for the Preservation and Management of Water Quality Samples” (HJ 493-2009), various samples are collected including field duplicate samples, full procedure blank samples, transport blank samples, and cleaning blank samples. The principle is that 10% of samples for each project are field duplicates, with at least one duplicate when fewer than ten samples are collected. When collecting field duplicates, samples are divided into equal volumes and preservatives are added. One full procedure blank sample, transport blank sample, and cleaning blank sample are set daily. After sampling, preservatives specified in HJ 493-2009 and HJ 494-2009 are added to the samples. Groundwater samples are immediately labeled and stored in a vehicle-mounted cooler below 4 °C for refrigerated transport. Samples are kept cool, away from light, and sealed.

#### Laboratory testing and quality control assurance

- Calibration Curves.
  For quantitative analysis, a calibration curve method is used with five standard solution concentration gradients (excluding blanks) that cover the range of the samples tested. The lowest concentration point is close to the method's detection limit. The correlation coefficient (r) of the calibration curve must meet the requirements of the testing standards. If the sample concentration exceeds the laboratory’s linear range of the curve, it is diluted and retested.

(b) Blank Experiments.
  A blank experiment is conducted with each batch of samples analyzed, ensure that at least one blank sample is tested per batch. The results of the project’s blank samples should be below the method detection limit or meet the testing standards for blank detection.

(c) Duplicate Samples.
  During each test, at least 10% of the samples collected in the field are duplicates, and at least 10% of duplicates are randomly drawn in the laboratory for quality control testing.

(d) Spiked Recovery.
  A specified concentration and volume of standard solution are added to the samples, which are then processed and tested like regular samples. Each batch must include at least one spiked sample, and the recovery rate for the project’s samples must be within the acceptable range of 80–120% of the testing standards.

(e) Quality Control Samples.
  Certified standard materials are tested using the complete testing procedure, and the test results must be within the standard range specified of the certificate.

## The statistical analysis of heavy metal pollution in underground water of mining areas

Before evaluating heavy metal pollution in underground water, it is essential to analyze the statistical characteristics and distribution patterns of indicator elements in the study area using statistical methods. Through statistical characteristic analysis, one can grasp the apparent features of heavy metal pollution in the underground water of the study area, offering the necessary basis for evaluating the results^23^. In this section, SPSS and Excel software were primarily employed to analyze the experimental detection data of selected heavy metal evaluation indicators, namely Mo, As, Co, Mn, Ba, Sb, and Se. The analysis mainly encompasses parameters like maximum value, minimum value, mean, variance, kurtosis, skewness, and coefficient of variation. This translation could be used for an academic paper.

The minimum and maximum values represent extreme values in the data, indicating the degree of heterogeneity within the dataset. The median is a type of central tendency that describes the typical scenario within a set of data. The mean represents a measure of central tendency within a dataset and serves as an indicator reflecting the trend of the data set. In statistical work, the mean (average) and standard deviation are the two most important measures used to describe the trend and dispersion of data sets. Variance and standard deviation are used to measure the deviation of a random variable from its expected value and are often employed to study the deviation between mathematical variables and their mean in various scenarios. The coefficient of variation, similar to variance and standard deviation, primarily indicates the degree of dispersion within data, influenced by both the dispersion level of variables and the average level of variable values. The statistical characteristic values of the indicator elements in the study area can be found in Table 2. This translation could be used in an academic paper.

**Table 2:** Statistical characteristics of heavy metal indicator factors.

	Mn	As	Ba	Co	Mo	Sb	Se
Range	0.110	0.650	0.057	0.0005	0.0090	15.362	0.019
Minimum	0.010	0.030	0.009	0.0001	0.0010	0.008	0.002
Maximum	0.120	0.680	0.066	0.0006	0.0100	15.370	0.021
Median	0.010	0.240	0.022	0.0001	0.0010	0.120	0.002
Mean	0.020	0.299	0.028	0.0002	0.0025	1.765	0.005
Std. Deviation	0.024	0.227	0.017	0.0002	0.0023	3.910	0.006
Variance	0.001	0.051	0	0	0	15.291	0
Skewness	3.338	0.232	0.987	1.617	1.840	2.540	1.727
Kurtosis	12.278	− 1.591	− 0.094	1.395	3.343	6.056	1.662
CV	119.87%	75.73%	58.52%	79.22%	92.04%	221.59%	111.12%
P.index	20.09%	2991.00%	4.05%	0.38%	3.57%	35,293.20%	53.76%
O.Std.index	3.85%	100%	0	0	0	100%	20%

(1) CV (Coefficient of Variation) = (Standard Deviation / Mean) × 100%; Standard values refer to the Drinking Water Standards (GB5749–2006);

P.index (Pollution Index) = Mean / Standard Value; O.Std.index (Exceedance Rate) = (Number of exceedances / Total monitored data points)×100%.

### Analysis of standard deviation characteristics of heavy metal evaluation index concentrations

From the results of the data analysis, it can be seen that the standard deviations for Mn, As, Ba, Co, Mo, Sb, and Se are 0.024081, 0.22651, 0.0166, 0.000152, 0.002301, 3.910359, and 0.006, respectively. The standard deviations for As and Sb are relatively large, with their maximum concentrations being 22.67 and 1921.25 times their minimum values, respectively. These results indicate a high degree of dispersion for Mn, As, and Sb in the sampled water, suggesting significant spatial variability of these two heavy metals in the groundwater of the study area, generally showing higher concentrations in the west and north, and lower in the east and south. This indicates that these two indicators have low background values and are greatly affected by mining activities in the antimony and iron mines of the region. This suggests that during the mineral smelting process, some equipment experiences issues such as leaks and spills, which significantly impact both surface water and groundwater in the mining area to varying degrees. The standard deviations of the other five ions are essentially zero, indicating that these ions mainly originate from natural background values. They are primarily due to the corresponding associated elements in the mineral components of the study area, which dissolve in the groundwater due to leaching actions and human factors, and are less affected by mining activities in the mining area.

### Analysis of kurtosis and skewness characteristics of heavy metal evaluation index concentrations

Under natural conditions, the concentration curves of heavy metal ions generally conform to a normal distribution. Kurtosis and skewness can reflect whether the concentration of a certain ion conforms to, approaches, or deviates from the norm. The degree of deviation can generally indicate whether the ion is influenced by anthropogenic factors. Kurtosis is a measure that reflects the shape of the distribution of a random variable. The higher the kurtosis, the more concentrated the values; the lower the kurtosis, the more dispersed the data. The kurtosis of a random variable x is defined as:

$$K = {\text{E}}[x - {\text{E}}(x)\left] {^{{4}} /} \right[{\text{Var}}(x)]^{{2}}$$

When K > 3, it represents a leptokurtic curve, indicating that the variable values are densely distributed around the mode.

When K = 3, it matches the degree of sharpness of a normal distribution curve.

When K < 3, it represents a platykurtic curve, suggesting a relatively uniform dispersion of variable values around the mode.

Skewness measures the degree of asymmetry and the direction of skew of a distribution. It is a dimensionless value. A higher skewness indicates that the values are generally larger than the mode of the distribution, while a lower skewness indicates that the values are generally smaller than the mode. The skewness of a random variable x is defined as:

$$S = {\text{E}}[x - {\text{E}}(x)\left] {_{{3}} /} \right[{\text{Var}}(x)]^{{{3}/{2}}}$$

When S > 0, the distribution of x is positively skewed.

When S = 0, it is symmetric around the mode.

When S < 0, the distribution of x is negatively skewed.

From Table 2, it can be seen that the kurtosis values for Mn, Mo, and Sb are 12.278 > 3,3.343 > 3, and 6.056 > 3, respectively, indicating that these metals are highly peaked relative to a normal distribution. On the other hand, the kurtosis values for As, Ba, Co, and Se are − 1.591, − 0.094, 1.395, and 1.662, respectively, all less than 3, indicating a flat-topped distribution curve. Among these, Mn, As, Ba, and Sb deviate significantly from the normal distribution curve. Based on water quality data, only three locations exceed the standard for Mn, while Ba does not exceed the standard at any location, suggesting that Mn and Ba are almost unaffected by anthropogenic factors and their concentration values primarily originate from natural background levels. As and Sb exceed standards at all locations, and their high peak values indicate that the water quality monitoring points for As and Sb in the mining area are severely affected by mineral extraction. The remaining three heavy metals have smaller peaks and are more dispersed, indicating different degrees of impact from human mining activities at various water quality monitoring points; the skewness values for all seven evaluated heavy metal ions are greater than 0, indicating positive skewness. Notably, Mn and Sb show higher skewness, suggesting that the analysis results reflect varying degrees of impact from mineral extraction on all water quality monitoring indicators in the study area.

### Analysis of statistical characteristics of heavy metal evaluation index concentrations

From the perspective of the coefficient of variation, the degree of variation among the seven factors is as follows: Sb > Mn > Se > Mo > Co > As > Ba, with the coefficients of variation for Sb, Mn, and Se exceeding 100%, and even 200%. The coefficient for Mn is notably high, primarily due to the significant influence of Mn ions in surface water bodies. The variability coefficient for Sb reaches 221.59%, mainly because the #2 monitoring point located upstream in the mining area groundwater is less affected by mineral extraction, whereas the remaining water quality monitoring points are affected to varying degrees, resulting in severe exceedances and thus a high variability coefficient for Sb. The smaller coefficient of variation for As is mainly because the water quality monitoring points in the study area are generally affected by the mining process, with all monitoring points showing severe exceedance for As. The pollution index for heavy metal indicators is in the order of Sb > As > Se > Mn > Ba > Mo > Co, with the pollution index for As reaching 2991% and for Sb as high as 35,293.2%, indicating severe pollution levels for antimony and arsenic from an average perspective. As shown in the table, the exceedance rates for As and Sb reach 100%, while Se and Mn have exceedance rates of 20% and 3.85% respectively, and the other three indicators have an exceedance rate of 0%. This indicates that phenomena such as leaks and spills occur during the smelting process of As and Sb elements due to mineral extraction.

### Analysis of pollution characteristics of heavy metal exceedances

**Figure 2:**
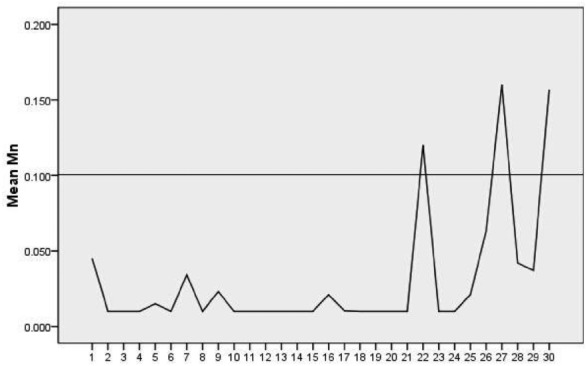
Current status of Mn concentrations.

To visually depict the pollution status of each sampling point's water samples in both groundwater and surface water for indicators showing exceedance rates, we have generated concentration status diagrams for the prevailing concentrations of Mn, As, Sb, and Se, which have non-zero exceedance rates. These diagrams illustrate a comparison between the concentrations of each exceedance factor against the standard values at each sampling point and depict the contrast among the sampling points, as shown in Fig. 2.

According to the Drinking Water Standard (GB5749-2006), the standard concentration for Mn is 0.1 mg/L. From Fig. 2, it's evident that only Spring 22 in Chuanshan Village of the mining area and surface water points DB1 and DB4 in Dongxia Village of the mining area and Shengli Village in Zhonglian Township respectively, exceed the standard concentration for Mn. Furthermore, it's notable that the Mn content in surface water is generally higher than in groundwater. Essentially, only point 22 exceeds the average Mn concentration in surface water, and only three groundwater sampling points exhibit Mn concentrations higher than those in surface water.

**Figure 3:**
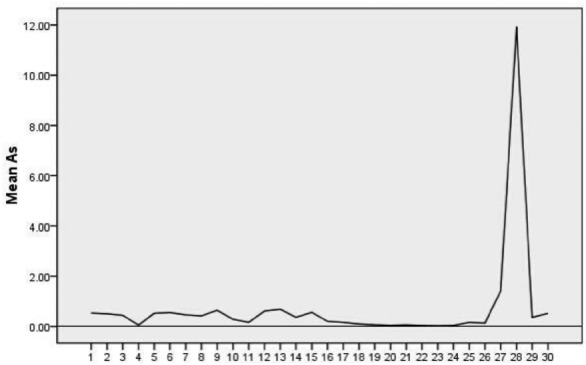
Current status of As concentrations.

Figure 3 illustrates that both surface water and groundwater within the study area exhibit arsenic (As) concentrations higher than the standard value of 0.01 mg/L for drinking water. Notably, the surface water point DB2 in Tanjia Community, Minshan Town, records an alarming concentration of 11.93 mg/L.

**Figure 4:**
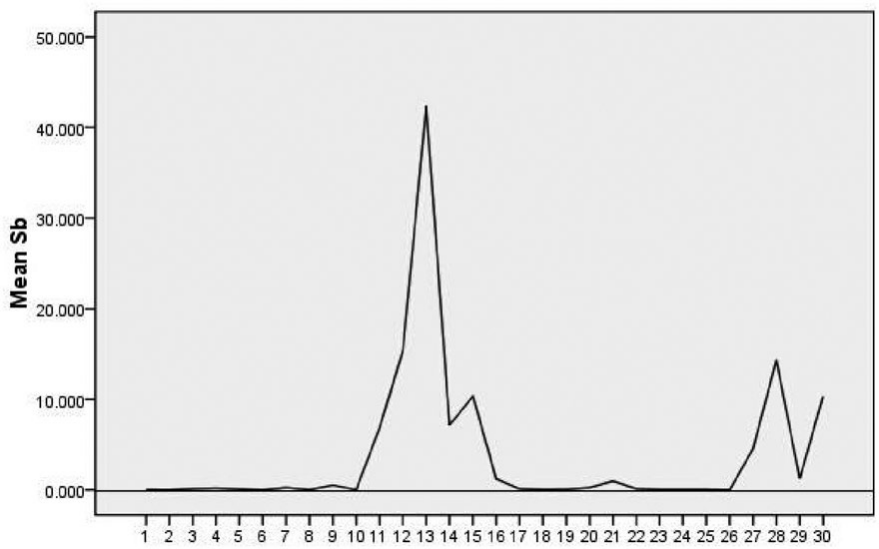
Current status of Sb concentrations.

Regarding antimony (Sb), after handling the outliers, spring water point 13 north of Minshan Hospital in Taotang Street, Minshan Town, was identified as an outlier and removed. Drainage from hospital facilities such as treatment rooms, laboratories, wards, laundry rooms, X-ray imaging, isotope therapy diagnostic rooms, and operating theaters intensifies the antimony pollution in the groundwater in that area, potentially compromising the accuracy of the water pollution assessment in the research area. This particular data point has been considered an outlier and excluded from the analysis. Figure 4 illustrates that the concentrations of Sb in all sampling points within the research area exceed the drinking water standard value of 0.005 mg/L, indicating severe antimony pollution. The minimum recorded concentration surpasses 0.0084 mg/L, more than 1.68 times the drinking water standard. Moreover, the Sb concentrations in four surface water sampling points across the research area are consistently higher than the median Sb concentration in groundwater.

**Figure 5:**
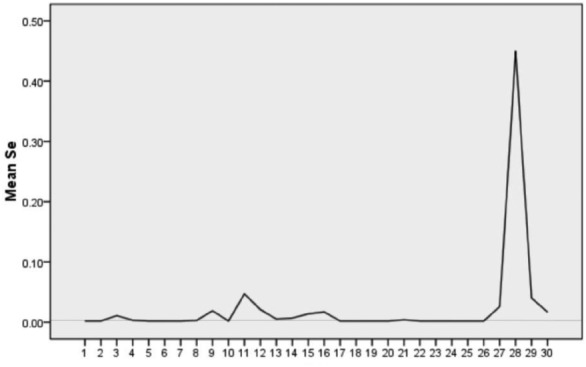
Current status of Se concentrations.

The standard value for selenium (Se) in drinking water is 0.01 mg/L in Fig. 5, and the exceedance rate at the groundwater monitoring points in the surveyed area is 20%. Water from the Qilijiang Iron Mine (11), in direct contact with the mine area, exhibits significant pollution with a concentration of 0.047 mg/L, considered an outlier. After removing this outlier, the analysis reveals some sampling points with selenium concentrations at 0 mg/L, while the maximum concentration reaches 0.021 mg/L, twice the standard value, suggesting a distinctly regional pattern of pollution. Overall, the analysis indicates that arsenic and antimony pollution is most severe in the groundwater of the study area, followed by selenium and manganese, with minimal contamination from barium, cobalt, and molybdenum.

## Principal component analysis of heavy metal elements

The abundant variety of mineral resources in the study area leads to a multitude of factors affecting the groundwater quality in this region. This paper utilizes SPSS software to conduct a principal component analysis on selected indicator factors. This approach, while minimizing data loss, involves linear combinations and discarding minor details, replacing multidimensional variables with a few comprehensive ones. This method aims to reduce workload while ensuring an accurate assessment of the groundwater quality in the study area.

### Principles of principal component analysis

Principal Component Analysis (PCA)^24–27^ is a statistical method for dimensionality reduction, aiming to quantitatively study multiple-dimensional factors within the same system. The fundamental concept lies in the assumption that among numerous correlated factors, there are dominant common factors. By analyzing the internal structural relationships within the correlation matrix of the original variables, this method identifies representative and targeted indicators, facilitating their mutual comparison. Additionally, it objectively determines the respective weight values, effectively capturing the main contradictions and significantly reducing subjectivity. In the evaluation of heavy metal pollution in groundwater, PCA proves highly persuasive, offering a robust mathematical model.

1 $$\begin{gathered} F1 = a11ZX1 + a21ZX2 + \ldots + an1ZXn \hfill \\ F2 = a12ZX1 + a22ZX2 + \ldots + an2ZXn \hfill \\ Fm = a1mZX1 + a2mZX2 + \ldots + anmZXn \hfill \\ \end{gathered}$$

Composite evaluation function:

2 $${\text{F}} = \frac{{{\uplambda }_{1} }}{{{\uplambda }_{1} + {\uplambda }_{2} + \ldots {\uplambda }_{{\text{p}}} }}{\text{F}}_{1} + \frac{{{\uplambda }_{2} }}{{{\uplambda }_{1} + {\uplambda }_{2} + \ldots {\uplambda }_{{\text{p}}} }}{\text{F}}_{2} + \frac{{{\uplambda }_{{\text{p}}} }}{{{\uplambda }_{1} + {\uplambda }_{2} + \ldots {\uplambda }_{{\text{p}}} }}{\text{F}}_{{\text{p}}}$$

where anm represents the eigenvectors corresponding to the eigenvalues of the covariance matrix of the original variable matrix X; ZX1, ZX2, ZXn are the standardized values of the original variable matrix X; λ1, λ2, …, λp represent the eigenvalues of the ZX matrix; n denotes the number of factors; m represents the number of samples; p stands for the number of principal components.

### Results and analysis

The analysis results of heavy metal indicator factors in water samples at sampling points using SPSS are shown in the Table 3.

**Table 3:** Evaluation index system correlation coefficient matrix eigenvalues and contribution rate.

Ingredient	Initial eigenvalue			Extracted principal components		
	Eigenvalue	Contribution rate %	Cumulative contribution %	Eigenvalue	Contribution rate %	Cumulative contribution %
1	2.902	41.463	41.463	2.902	41.463	41.463
2	1.306	18.650	60.113	1.306	18.650	60.113
3	1.119	15.979	76.093	1.119	15.979	76.093
4	0.840	12.000	88.093			
5	0.361	5.159	93.252			
6	0.284	4.053	97.305			
7	0.189	2.695	100.000			

From the Table 4, when the cumulative contribution rate is greater than or equal to 75%, three main components can be extracted. The eigenvalue of the first principal component is 2.902, the second principal component is 1.306, and the third principal component is 1.119. The contribution rate of each eigenvalue represents the weight of each principal component, i.e., λ1 = 41.463%, λ2 = 18.65%, λ3 = 15.979%. Therefore, three principal components are extracted, and a principal component matrix is established.

**Table 4:** Principal component matrix.

Principal Components			
Factor	1	2	3
Mn	− 0.302	0.083	0.613
As	0.703	− 0.538	− 0.013
Ba	0.583	0.750	− 0.067
Co	0.358	− 0.119	− 0.746
Mo	0.660	− 0.554	0.273
Sb	0.840	0.337	0.059
Se	0.841	0.108	0.322

The initial factor loading matrix displays the correlation coefficient values of each principal component with its corresponding variables. In the principal component matrix, dividing each column vector of the mth component by the square root of the mth eigenvalue provides the coefficients corresponding to each indicator for the mth principal component, i.e., the eigenvectors a1, a2, and a3, as shown in Table 5.

**Table 5:** Eigenvectors of the correlation matrix.

Eigenvectors	X1	X2	X3	X4	X5	X6	X7
a1	− 0.177	0.413	0.342	0.21	0.387	0.493	0.493
a2	0.073	− 0.47	0.656	− 0.104	− s0.485	0.295	0.095
a3	0.579	− 0.012	− 0.063	− 0.705	0.258	0.056	0.304

The eigenvectors obtained from Table 5 were multiplied by the standardized variables of the seven original factors to obtain the principal components.

$$\begin{gathered} {\text{F1}} = - 0.{177}*{\text{ZMn}} + 0.{413}*{\text{ZAs}} + 0.{342}*{\text{ZBa}} + 0.{21}*{\text{ZCo}} + 0.{387}*{\text{ZMo}} + 0.{493}*{\text{ZSb}} + 0.{493}*{\text{ZSe}} \hfill \\ {\text{ F2}} = 0.0{73}*{\text{ZMn}} + - 0.{47}*{\text{ZAs}} + 0.{656}*{\text{ZBa}} + - 0.{1}0{4}*{\text{ZCo}} + - 0.{485}*{\text{ZMo}} + 0.{295}*{\text{ZSb}} + 0.0{95}*{\text{ZSe}} \hfill \\ {\text{F3}} = 0.{579}*{\text{ZMn}} + - 0.0{12}*{\text{ZAs}} + - 0.0{63}*{\text{ZBa}} + - 0.{7}0{5}*{\text{ZCo}} + 0.{258}*{\text{ZMo}} + 0.0{56}*{\text{ZSb}} + 0.{3}0{4}*{\text{ZSe}} \hfill \\ \end{gathered}$$

The comprehensive assessment index was calculated from the principal component functions and their corresponding eigenvalues, as shown in Table 6.

**Table 6:** Principal component analysis results.

Sampling point	First principal component F1	Second principal component F2	Third principal component F3	Comprehensive principal component F	Ranking
11	5.419	− 0.805	− 2.913	5.498	1
12	3.719	0.422	0.545	2.866	2
15	3.205	0.178	1.668	2.186	3
13	4.099	2.385	3.044	2.069	4
9	1.828	0.033	− 0.366	1.62	5
16	0.839	0.121	− 0.732	0.873	6
3	0.375	− 1.086	− 0.167	0.6	7
14	1.249	0.678	1.619	0.454	8
1	− 0.239	− 0.853	− 0.128	0.024	9
21	0.056	1.198	− 0.796	− 0.003	10
20	0.135	1.454	− 0.681	− 0.025	11
2	− 0.367	− 2.291	0.916	− 0.046	12
10	− 0.726	− 1.366	0.659	− 0.482	13
5	− 0.689	− 0.461	0.149	− 0.514	14
8	− 0.765	− 1.774	1.186	− 0.567	15
6	− 1	− 0.53	0.362	− 0.816	16
24	− 1.327	0.393	− 0.657	− 1.018	17
19	− 1.44	0.233	− 0.644	− 1.081	18
04	− 1.407	− 0.217	− 0.156	− 1.086	19
18	− 1.443	0.335	− 0.211	− 1.223	20
7	− 1.566	− 0.517	0.4544	− 1.317	21
23	− 1.836	0.115	− 0.509	− 1.422	22
17	− 1.772	0.004	0.018	− 1.487	23
25	− 1.887	0.019	− 0.275	− 1.506	24
22	− 1.954	1.994	− 1.547	− 1.656	25
26	− 2.502	0.339	− 0.839	− 1.939	26

1. According to Table 3, the eigenvalues of the first, second, and third principal components are 2.902, 1.306, and 1.119 respectively. The contribution rates of the principal components are 41.463%, 18.650%, and 15.979% respectively, accumulating to 76.093%. This indicates that these three principal components essentially encompass the influencing factors of heavy metal pollution in the groundwater of the research area. The first principal component, in particular, contains more information and exhibits a stronger correlation with water quality.
2. From Table 5, it can be seen that the first principal component, a1, has a high correlation with the original variables Sb and Se, reaching 0.493. This suggests that these two indicator factors may originate from the same pollution source and are the main heavy metal pollutants of this pollution type, likely due to improper disposal of mineral smelting wastewater or slag. The contribution rates of the other five indicators to pollution are smaller. The second principal component, a2, shows a strong positive correlation with Ba in the original variables, reaching 0.656, and a strong negative correlation with Mo, at − 0.485. This indicates that Ba and Mo make significant contributions to this component, primarily influenced by the leaching action of minerals, with Ba and Mo exhibiting an inverse relationship. The third principal component, a3, has a strong positive correlation with Mn in the original variables, at 0.579, and a strong negative correlation with Co, at − 0.705. This shows that Mn and Co have significant contributions within a3, mainly influenced by the leaching effect of the ore’s chemical composition, and Mn and Co may engage in cation exchange interactions.
3. Based on the comprehensive principal component score F, Table 6 is obtained. A higher F value indicates more severe heavy metal pollution. Among these, sampling points 11, 12, and15 rank in the top three, signifying the most severe heavy metal pollution at these locations. On the other hand, sampling points 25, 22, and 26 rank at the bottom three, indicating the least polluted water samples among the research sampling points.
4. The distribution of heavy metal pollution indicates that the less contaminated monitoring points, such as points 26, 22, and 25, are all located in the upstream area of the groundwater. The most severely contaminated sites are mainly concentrated in the central part of the mining area or the downstream region of the groundwater. This suggests that the background values of heavy metal ions in the survey area are relatively low, and the main sources of heavy metal ions in the groundwater of the mining area and its surroundings are derived from the mining of tin, antimony, and iron ores. In both the northern and southern mining areas, the majority of waste rock slag is disorderly piled up, and the improper disposal of antimony slag after smelting activities—lacking lining and covering measures—allows ongoing acidic reactions from atmospheric rainfall infiltration into the slag piles, releasing heavy metals into the groundwater. Additionally, the discharge of mine pit water and smelting wastewater, along with the infiltration of rainwater, further accelerates the leaching of contaminants into the groundwater. These factors are the main pathways for the contamination of groundwater with heavy metals such as arsenic and antimony in the mining area, as evidenced by the significantly higher antimony pollution around the antimony slag compared to the surrounding areas.

## Conclusion

1. Among the evaluation indicator factors of groundwater monitoring points in the research area, the standard deviation analysis reflects that As and Sb are more sensitive to the influence of mining activities. Mn, Se, Mo, Co, and Ba are primarily influenced by natural geological and hydrogeological conditions, with less impact from human activities. Skewness and kurtosis values of heavy metal evaluation indicators reveal varying degrees of anthropogenic environmental impact on the seven factors. The pollution sources of Mn, Mo, and Sb are relatively concentrated, while those of As, Ba, Co, and Se are more dispersed. The exceedance rate for As and Sb reaches 100%, with Se and Mn exceeding by 20% and 3.85%, respectively, while the exceedance rate for the remaining three indicator factors is 0.
2. The severity of pollution in the research area's groundwater is highest for As and Sb, followed by Se and Mn. In contrast, Ba, Co, and Mo's pollution levels among these three heavy metals have not significantly surpassed drinking water standards.
3. Through principal component analysis, the first principal component a1 exhibits a higher correlation of 0.493 with the original variables Sb and Se. The second principal component a2 shows a strong positive correlation of 0.656 with the original variable Ba and a notable negative correlation of − 0.485 with Mo. The third principal component a3 displays a positive correlation of 0.579 with the original variable Mn and a strong negative correlation of − 0.705 with Co.
4. In summary, the heavy metal pollution in the antimony mining area of Lengshuijiang City, Hunan, is in a very serious state, with significant over-standard levels of the heavy metals arsenic (As) and antimony (Sb). The main cause of this situation is the improper stacking and disposal of mining waste and wastewater. When rainwater infiltrates these waste piles, it causes the release of heavy metals into the groundwater. Additionally, the discharge of mine pit water and smelting wastewater, along with the percolation of rainwater, further accelerates the leaching of contaminants into the groundwater. Therefore, it is urgent to address the standardization and remediation of the mining area as well as the restoration of groundwater. Although research on heavy metal pollution management has been increasing annually, the management of groundwater heavy metal pollution remains a challenging issue. Only by cutting off the source can further aggravation of groundwater pollution in the mining area be prevented.

## Data Availability

The data have been explained in the paper.
